# Approaching Trust: Case Studies for Developing Global Research Infrastructures

**DOI:** 10.3389/frma.2021.746514

**Published:** 2021-11-01

**Authors:** Heather Flanagan, Laurel L. Haak, Laura Dorival Paglione

**Affiliations:** ^1^ Spherical Cow Consulting, Vashon, WA, United States; ^2^ Ronin Institute, New York, NY, United States; ^3^ Mighty Red Barn, Townsend, WI, United States; ^4^ Laura Paglione, LLC, Rego Park, NY, United States

**Keywords:** multi stakeholder initiative, trust and reciprocity, stakeholder theory (normative foundations), public infrastructure, IT governance (ITG)

## Abstract

Trust is a core component of collaboration. Trust is a local phenomenon, and scientific research is a global collaborative, its impact multiplied through open exchange, communication and mobility of people and information. Given the diversity of participants, local policies and cultures, how can trust be established in and between research communities? You need transparent governance processes, thoughtful engagement of stakeholder groups, and open and durable information sharing to build the “stickiness” needed. In this paper we illustrate these concepts through three trust building use cases: ORCID, Global Alliance for Genomics and Health, and SeamlessAccess, platforms sharing an identity and access technical service core, painstaking community building, and transparent governance frameworks.

## 1 Introduction

Research is a global endeavor of iteration and collaboration. Research requires trust-building: shared understanding of process, access to source data, and points of validation. A number of trust structures are used by researchers: disciplinary societies cohere practices among researchers, educational degrees and institutional affiliation are proxies of trust, as is publication of research findings in status journals (Haak and Wagner, 2021).

These trust structures require interactions among many stakeholder groups, operating within and across disciplines, institutions, and countries. This is where research infrastructures come into play. These infrastructures support knowledge sharing across stakeholder borders, and at the best of times create a foundation for collaboration (Edwards et al., 2013; Haak et al., 2020). Examples of global-scale research infrastructures include article indexing platforms, researcher profile systems, federated identity systems, data repositories, and global data collection systems. More recently, the research community has started to pay more interest to the governance and sustainability aspects of these infrastructures (Bilder et al., 2020; Skinner, 2019). Organizations such as the Research Data Alliance have fostered cross-disciplinary self-organization of community stakeholders, out of which have come truly amazing consensus rules of behavior—principles of findability and accessibility (Wilkinson et al., 2016), as well as responsibility and ethics (Carroll et al., 2020)—that can be applied to infrastructures to improve research rigor and reproducibility and ultimately improve trust and engagement in the research process.

In this article, we share our “in the trenches” experiences of how these principles, when applied in practice, can drive research infrastructure adoption. Infrastructure is more than a platform, it is a public good, so we need to ensure its accessibility and sustainability. How it is constructed, governed, and maintained requires intentional engagement and alignment of diverse stakeholders across social and economic factors to maximize trust, utility and impact on public welfare (Dhanshyam and Srivastava, 2021). What we have found is that without alignment and engagement, trust-building suffers. The lower the trust—even for a really strong technology that is desperately needed by the research community—the steeper the uphill push to adopt and implement the infrastructure.

## 2 Approach

Infrastructure adoption depends on how well it serves its intended audience. There are multiple factors involved in building the trust needed for adoption: identification of stakeholders, development of services that respect and meet the needs of these stakeholders, governance (including openness and transparency), communications and marketing, start-up funding, and sustainability (including processes and recurring funding). In this article, we explore stakeholder alignment and engagement as fundamental components of trust-building. We take an ethnographic approach, which involves an emphasis on the “emic” or insider view, rendered as first-person case-study accounts (Eriksson and Kovalainen, 2014). This methodology has been used effectively in studying development of new-technology-based services (Eriksson et al., 2011).

We chose research infrastructure initiatives that share a core of painstaking multi stakeholder community engagement around individual privacy, and also that illustrate the impact of different stakeholder economic and social motivations on alignment and engagement, and, ultimately, on infrastructure adoption. We each have been intensively engaged in the formation of the global-scale research infrastructures SeamlessAccess, Global Alliance for Genomics and Health, and ORCID. Here we share our experiences and impressions of their early-stage development.

### 2.1 SeamlessAccess

Heather Flanagan was the Pilot Coordinator for the RA21 Academic Pilots and, later, served as Program Director for the follow-on effort, SeamlessAccess. She brought to the table expertise in federated identity and a strong network in the academic identity federation community, supporting both community engagement and technical specifications work. Laure was engaged as a stakeholder, providing input on multi-stakeholder governance principles. Laura was engaged as a subject matter expert on researcher privacy and end-user design. (Note that business ethnography often recommends that researchers embed into their research projects as team members, taking on roles such as project manager, to gain oversight into all aspects of an initiative without necessarily shaping it.) The case study was shared with the SeamlessAccess management team and their comments have been incorporated into the narrative.

### 2.2 Global Alliance for Genomics and Health

Laura Paglione was engaged with the Data Use and Researcher Identity working group as a volunteer and subject matter expert on researcher identifiers and federated identity. She also served as the Co-chair of the Equity, Inclusion, and Diversity Advisory Group that has a goal to understand, encourage, and support broad participation in the volunteer groups participating in this effort. At the leadership level, she made connections between global standards initiatives and community engagement approaches. The flavor of working group discussions is reflected in the storytelling approach and metaphorical examples used in this case study.

### 2.3 ORCID

Laurel Haak was the founding Executive Director of ORCID, and Laura Paglione its founding Technical Director, employees 1 and 2, respectively. Both were deeply engaged in starting up organizational operations, establishing participatory co-design culture, engaging stakeholders from pre-launch to implementation and through to specifying initial versions of a certification program, and were principal architects of the ORCID Trust program. Perspectives of ORCID team members and stakeholders are incorporated by reference to blog posts and primary documents.

## 3 Manuscript

### 3.1 Case Study: SeamlessAccess

Trust is local. How do you then approach building a global information technology service? How do you make the work local enough for global participants to trust the outcomes of the collaboration? How do you manage expectations when the technology is too complex for anyone other than experts to understand? A number of studies in public sector organizations show that transparent governance processes and thoughtful invitations to key stakeholder groups are key to the adoption and sustainability of information technology (Wiedenhöft et al., 2020). Trust can generate a certain “stickiness” when individuals feel their community has been heard (AlHogail, 2018), but it depends on ongoing and open engagement within and between stakeholders.

In this case study, we explore how building trust is complicated by poorly understood technology, uncertainty regarding ownership, and loosely aligned stakeholders. We will also look at the additional complexities of rebuilding broken trust between stakeholder communities.

#### 3.1.1 Introducing SeamlessAccess

Technology introduces a wonderful world of online access opportunities. Early adopters, however, often find that the user experience is an afterthought to the technical implementation of an idea. One example of this is the world of federated identity, which offers students and researchers the ability to log in (authenticate) to an online service (such as a library catalog) via the credentials (such as their username and password) managed by their home organization (such as their college, university, or company), also known as an Identity Provider. There is quite a bit of value here: users do not need to remember yet another password, services do not need to maintain user affiliation records, services can enable sign in from multiple organizations at once through Identity Provider federations, and the impact of account compromise within the service provider is limited. Federated identity has been available, particularly in academia, for over two decades.

Unfortunately, while federated identity offers powerful benefits, the complicated user experience associated with the technology has caused many scholarly communications services—particularly publishers—to avoid implementing it. In 2015, however, publishers decided that the benefits of federated identity were great enough to warrant addressing the user experience. Publishers were feeling pain on multiple levels. A long-established process of maintaining lists of customer IP addresses to indicate which organization the user was from was becoming untenable because they were no longer the stable data they were before computing went mobile. Publishers heard demands directly from users to improve off-campus access to content (away from campus IP addresses). And, publishers were experiencing a business model threat as pirated material became more easily accessed than legally provided content.

These factors led to a community collaboration called “Resource Access in the Twenty-First Century” (RA21), and then later to an operational service called SeamlessAccess (NISO, 2019). RA21 focused on developing “recommendations for using federated identity as an access model and improving the federated authentication user experience.” Those recommendations led to the creation of an operational service: SeamlessAccess. SeamlessAccess, in turn, provides a significantly improved user experience for helping users find their Identity Provider in a sea of options when trying to log in to a website that supports federated authentication.

RA21 identified business needs and user demand for a change in accessing scholarly content online. However, the demand for change was not sufficient to encourage trust in the thing that promises that change. The stakeholders involved—the scholarly publishing and the academic library communities in particular—were aligned on neither the problem that needed to be solved nor the solutions possible to solve it.

RA21 and later SeamlessAccess were driven largely (but not entirely) by the scholarly publishing community, in collaboration with researchers and campus IT. These relationships are reflected in the current SeamlessAccess governance body: a coalition of GÉANT, Internet2, the National Information Standards Organization (NISO), and the International Association of STM Publishers. Publishers were hearing from their readership and from the researchers on their editorial boards that access methods needed to change. Publishers engaged with the federated identity and campus library communities, but publishers and campus IT tend to exist in tension with the library community—largely because of differing perspectives on content access. A particular issue for librarians was concern about the impact a move to federated identity might have on a user’s right to privacy. In addition, given ongoing budget challenges, librarians had little desire to argue within their own organizations for the resources necessary to support implementation of federated identity. Stakeholders in the scholarly communications ecosystem were not aligned.

#### 3.1.2 Components of Trust

When working with technology, trust comes from more than understanding the technology itself. It also requires trust in how the service that uses the technology is managed and how the service engages with its stakeholders. And of course, each piece - technical understanding, governance, and stakeholder engagement - is interdependent. SeamlessAccess has focused on stakeholder engagement as the core of its trust model, out of which comes technical requirements and governance decisions.

##### 3.1.2.1 Technical Understanding

“Any sufficiently advanced technology is indistinguishable from magic.”—Arthur C. Clarke.

A century ago, business owners probably looked askance at this newfangled thing called an automobile. They likely wondered how they were supposed to let their business depend on this new thing when they had no idea how it even worked, how to maintain it, or how to even pay for it. Eventually, the infrastructure matured enough to make the automobile the ubiquitous thing it is today. Federated identity is also in that early stage where the infrastructure isn’t ubiquitous enough that people are just willing to trust that it works.

Like an automobile, federated access is quite complicated under the hood. The trick is to help stakeholders understand what it can do and how it can help solve their problems, without digging too deep into the technology details. There’s a caveat to that, however: while you don’t want to overwhelm stakeholders with unnecessary detail, that detail must be available for those who want to learn more. The transparency of the technology is critical, while understanding the technology is not.

SeamlessAccess has focused much of its efforts on building an outreach program to educate different stakeholders about federated identity. The Outreach Committee in particular brings in representatives from the primary stakeholder groups to maintain perspective on what kinds of questions people are asking, and determine how to speak to their concerns. The purpose here is to effectively translate the technology for the understanding and benefit of end users.

But there’s another component: the technology is not beneficial if it is not implemented. Prior to RA21 and SeamlessAccess, there was no standard way to present federated access to the user. Every service presented the information in different, and often very confusing, ways. On the one hand, SeamlessAccess exists to improve that user experience. On the other hand, the service relies on the organizations integrating it to offer feedback on what’s working for their users and what isn’t. To this end, SeamlessAccess is helping organizations that want to implement the technology understand what to do and, as importantly, why to do it that way. Allowing for the flexibility for integrators to experiment a bit with what will work best for them is another way to encourage trust in the service. The service has been tagged as a ‘beta’ product because changes are expected as we iterate on user testing and integrator feedback.

##### 3.1.2.2 Governance

Governance is how community projects make decisions that represent stakeholder needs, wants, and desires. A trusted governance model requires that each stakeholder must see someone in the governance group, either a person or an organization, that they can identify with. They must also understand the motivations and business model behind the service. Is the service for profit? If so, who is making money off of it? Or is the service not-for-profit? If so, how is it being sustained?

In the case of SeamlessAccess, governance happens in layers. There is a core governance team that focuses on legal and financial details, such as developing the by-laws needed to make the project a not-for-profit legal entity. This core group reflects the diverse stakeholder communities that will use the service and the organizations providing the resources to support the operations of the service. Next, there are several committees, including an Advisory Committee, an Outreach Committee, and a Technical Steering Committee. And third, there are working groups to focus on specific challenges, including the Contract Language Working Group and the WAYF Entry Disambiguation Working Group. This layered approach increases the opportunities for people and organizations to engage at a level comfortable to them. The more ways to actively engage stakeholders in governance, the more opportunities to build community trust in the service. The community needs to see that all parties working towards a common goal - building and maintaining a service that benefits all stakeholders.

##### 3.1.2.3 Stakeholder Engagement

The RA21 project polarized the library community, between supporters of federated identity who saw it as a way to enable access to content, and detractors who felt federated identity would lead to a loss of user privacy. SeamlessAccess inherited some, if not all, of that tension. Knowing that trust-building is a core success factor for this project and is a key aspect of its sustainability, the SeamlessAccess governance group worked to engage library stakeholders in design and governance discussions and made it a priority to offer additional education about federated identity.

It is worth noting that stakeholder tension was not restricted to publishers and librarians. Another tension that impacted efforts to promote federated identity as a solution for remote access to content was the tension between campus librarians and campus IT departments. Many librarians have existing infrastructure that lets them manage access to content. Shifting to a federated authentication model would require deeper collaboration between the library and campus IT. These two departments usually exist in entirely different parts of the campus organizational structure, with different priorities, funding, workflows, and users to support. Relations between campus libraries and IT are often weak at best, and antagonistic at worst.

###### 3.1.2.3.1 Getting Stakeholders to the Table.

With so many stakeholders, building trust starts with one of the most difficult steps: getting all the stakeholders to the table. If a stakeholder group is not willing to have a conversation, building trust with that group is simply impossible. Of course, once the groups are at the table, there needs to be a concerted effort to build credibility and understanding. Why should anyone at the table trust the convener, much less anyone else participating? NISO and the Research Data Alliance have been particularly effective in this arena (Carpenter and Horton, 2012). A shining example of cross-stakeholder trust building in the scholarly community is the Scholix Initiative, which engages across campus, governmental agencies, identifier infrastructures, data centers and publishers to create an internationally-used method to link research data with the literature (Cousijn et al., 2019).

###### 3.1.2.3.2 Inheriting Stakeholder Dependencies.

In the case of SeamlessAccess, to establish trust required bringing together stakeholders who understood the global federated identity infrastructure, people who understood the demands on campus IT, representatives from content providers, and content stewards. All were needed to build the service. However, these stakeholders can sometimes be only indirectly impacted by the value of the service offered. When the service needs the stakeholders more than the stakeholders need the service, it creates lopsided value. This has been a particular issue for SeamlessAccess.

For SeamlessAccess to function, the library itself or its campus needs to be a member of an identity federation. Campus IT and identity federations themselves are not directly impacted by the service SeamlessAccess offers. They have no pain point that this service can directly resolve; they have less motivation to come to the table. And yet, without their cooperation, federated identity is not possible. Without federated identity, there is no SeamlessAccess service.

###### 3.1.2.3.3 But what About the User?

As with many technology-based projects, there are many stakeholders who need to have a say in how the project progresses. SeamlessAccess is about creating a better experience for the individual actually trying to access protected content. So, where is the individual? Who represents them? While they are at the forefront of consideration, they do not have a direct seat at the table. The responsibility for representing the individual falls to the other stakeholders involved in the project.

Individuals are the most difficult group to engage with because they cannot be easily described in a way that would allow any one person or organization to represent all their needs. On a single campus, an individual user could be an undergraduate student, a graduate student, a faculty member, a researcher, a staff person, a contractor, a visiting student, a visiting scholar, or even a walk-in patron to the library. They all have different perspectives and may have entirely different needs when it comes to technology.

In the SeamlessAccess scenario, many of the stakeholders laid claim to being the representative for the individual user. Despite that representation, the needs identified are still in conflict; the stakeholders each see one aspect of a very complex ecosystem, resulting in perfectly valid needs that are in conflict with each other. Libraries staunchly defend privacy rights for the user in the face of users regularly giving away information about themselves if that’s the easiest way to get to the material they need. Campus IT has technical control but not authoritative control over attributes released by the campus. Publishers interact directly with the user and have an entirely different perspective on personal data collection. They are all accurate, even when they are in conflict.

###### 3.1.2.3.4 Building Trust Through Engagement.

**FIGURE 1:**
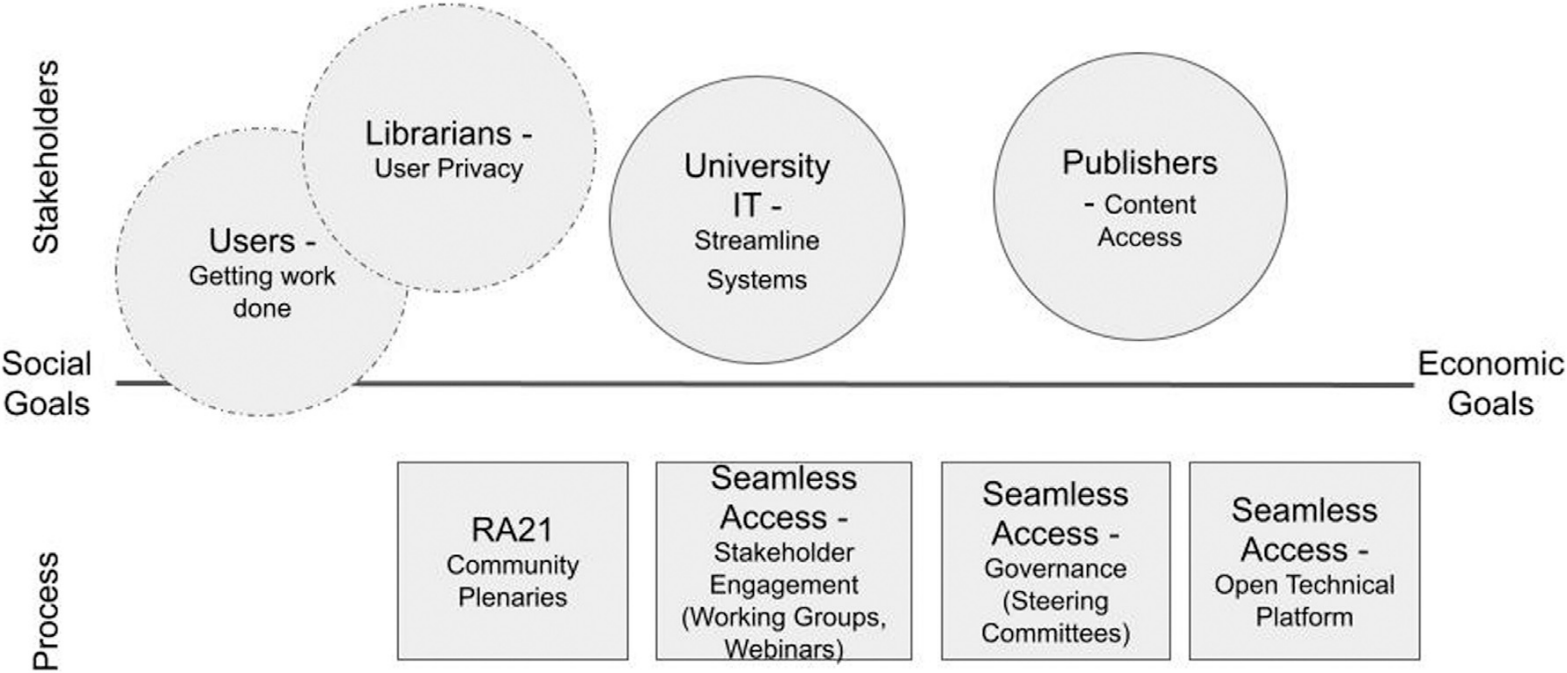
RA21 and Seamless Access were driven by publishers, who have an economic incentive to improve content access. The initiatives employed community outreach tactics and developed a technical platform, but did not have a transparent multi-stakeholder governance process for deciding on requirements.

Meaningful engagement efforts will slowly erode distrust between stakeholders and build trust in the service offering. In an ideal world, as the stakeholder groups see each other engage in good faith, and users benefit from the service, we will see tension that may exist between groups decrease. By educating and explaining what technology can and cannot do—and by ensuring stakeholders a seat at the decision-making table—it is possible to bridge the fact that sometimes, stakeholders simply do not trust the intent of other stakeholders (Figure 1).

Of course, the world is rarely an ideal place, particularly over the last year. As conferences and in-person meetings became a thing of the past, opportunities to let food, drink, and a full view of a person’s body language smooth human engagement have not been an option. SeamlessAccess has worked to engage stakeholders at virtual conferences and webinars, through targeted white papers and short videos explaining the service. We also collectively recognized that users were no longer able to live and study on campus and had to have better solutions for remote access. Members of various stakeholder communities that were not bought into the premise of federated access have experienced the need in ways they never have before. Suddenly, there has been a much stronger motivation to find ways to trust the technology, the other stakeholders, and the service itself.

#### 3.1.3 Learnings

SeamlessAccess focused on four challenges when considering how to build trust in the service:

• The service relies on complex technology and yet we need to make it transparent and trustworthy without expecting everyone to understand the details.
• Given the service is still under development, the user value may not be clear to everyone and is subject to change.
• The service needs the stakeholders more than the stakeholders need the service; it creates a lopsided value proposition.
• The service operators do not have direct access to the end-users, resulting in second-hand interpretations from various stakeholders on user needs.

We have addressed these options by focusing on educational outreach opportunities, a layered governance model, and strong stakeholder engagement. Each of these activities will require continued action to maintain the trust we’ve built and to continue to grow trust, and through it, service adoption.

### 3.2 Case Study: Global Alliance for Genomic Health

It’s 8:00 AM eastern on Wednesday morning, and the Data Use and Researcher Identity (DURI) work stream of the Global Alliance for Genomic Health (GA4GH) is meeting virtually. The group meets every 6 weeks to develop standards for computers to facilitate researcher access to genomics datasets. Today they are working on a standard for computers to understand if the person signing into the system is considered to be a bona fide researcher.

The systems in question are sensitive ones. They house genomics data that has been compiled for research purposes. Access to these data is restricted. Data Access Committees (DACs) review requests for access to the data, weighing information about the person requesting access (the data user) and the type of study being conducted (the study topic). The information in these datasets is de-identified so that it is not possible to tie it to the specific people (genetic data contributors) from which it was collected or derived. In addition, contributors whose genomic data are (or are not) included in datasets have control over the use of their data. For example, contributors may restrict the types of studies or diseases/conditions for which their data can be used, or if their data are used at all.

There are challenges. The process includes an inefficient process of collecting and exchanging information about the rules associated with each entry into the dataset. It also requires the need for checking each data user’s credentials to ensure that only people with appropriate researcher credentials are granted access.

On this morning, the DURI work stream was considering one part of this puzzle: creating a standard to streamline the credential check at the moment when a person attempts to access a dataset. What credentials should this person have to gain access? Can only bona fide researchers access the data or are there other types of experience that can qualify a person as a data user?

The work stream group consists of identity and access experts from around the world, a unique and finely refined group. They hail from technology companies, data repositories and globally-recognized research institutions. They understand the technology involved and its application to sensitive datasets like those in health care settings. They also understand how the technical components are very much an extension of prior work, knowledge obtained from a career-worth of education and experience. Because the group members have compatible and similar backgrounds, they don’t have to start from basic principles to develop this standard. Instead they can rely on their shared experiences from doing similar work.

In this case study, we explore how trust is impacted by including a diverse group of people early in the process, and how practical feedback and iteration using work outputs greatly enhances adoption.

#### 3.2.1 The Expert Conundrum

We need experts to create standards, policies, specifications, and research-based findings, but it is exactly this approach that can lead to a lack of trust among those who need to reference, use, or be governed by these outputs. These experts often have similar backgrounds and experiences, and share an understanding of the “prior art” on which they are building.

However, the community for which the experts are creating standards for almost always includes individuals that do not have this underlying understanding or experience. Without it, the work created by the experts can appear disjointed, illogical, and confusing, and these conditions can create a communication gap in explaining and understanding the standard. This gap can lead to mistrust.

#### 3.2.2 Experts Doing Expert-y Things

The participants of the DURI work stream that Wednesday morning were talking about how information (credentials) about the person requesting access should be passed from system to system. Since the members of the group all came from software backgrounds, they were familiar with several protocols for keeping information secure while transferring it to other systems. They also understood existing standards for managing user sign-in to a particular system, and could take for granted that all involved could understand the contents of a written technical standard that described a technical method for ensuring that exchanged information hasn’t been tampered with. A simple reference “shorthand” to this tacit knowledge is all that would be included to describe this implicit knowledge in the new standard being developed. There was no need to explicitly describe it because all involved in writing the standard would understand that these additional considerations were included in creating the standard; the “shorthand” (maybe a statement as simple as “using industry-accepted standards”) would be enough to assure those with similar background that these items were considered and handled. But how is trust and understanding engendered for those without this background?

At times, the work done by a group of experts is so technically specific that the group doing the work can establish trust based on the “truths of the field.” For example, trust in a computer network standard (and those who create it) may be established through an examination of the network’s technical capabilities and the feasibility of equipment to accomplish the stated goal. In these situations, the group creating the technical specifications, defining standards, and building policies are subject matter experts in the topic, an elite group that tends to be somewhat homogeneous in background, training, values (at least in this topic area), and approach (which is often rooted in the discipline). This condition can lead to more efficient work, as the norms, approach, and “givens” for the area are often already negotiated and accepted.

But, those using or affected by the standard may be more skeptical. The “shorthand” that is so effectively used among the group of experts may be off-putting and “jargon-y.” If trust has not already been established between stakeholders—data users, data contributors, and experts—, the data users and contributors can grow suspicious of whether the experts are acting in the end user’s best interest (Petts, 2008).

#### 3.2.3 The Impact on Trust

“We need to be willing to risk embarrassment, ask silly questions, surround ourselves with people who don’t know what we’re talking about. We need to leave behind the safety of our expertise.”—Jonah Lehrer, Imagine: How Creativity Works.

It is critically important that a technical standard be well understood and trusted by technical experts. These individuals have the background and expertise to be able to determine the quality and effectiveness of the standard. But too often technical standards teams nearly exclusively consider technical experts when gathering input for or communicating outputs about standards, often at the expense or exclusion of other stakeholders. This approach can have a very real impact on the trust that the other audiences put in the standard. This trust gap could ultimately impact the standard’s adoption success. In addition, the absence of engagement could provide an information vacuum that may be filled by misinformation, further jeopardizing adoption.

#### 3.2.4 Who Is the Expert?

Who should we consider to be the “expert” in these types of efforts? You, someone who may be reading this article in a published journal, may consider the scholarly community to be the experts—those who have studied the field, researched the impacts, and analyzed the data. But, this lens may not be broad enough to quell doubt, engender trust, and produce acceptance (Noordegraaf, 2020).

In 2021, familiar objections to vaccines (History of Anti-vaccination Movements, 2018) resurfaced as governments and institutions prioritized vaccines as a way to return to pre-pandemic activities after COVID-19. The points of objection to the COVID vaccinations mirrored historic ones often rooted in fear, misunderstanding of the science or source and contents of vaccine raw materials, concerns about reduction of personal liberties, and suspicion of the intentions of those advocating for the vaccines. The net impact of these objections is a lack of trust in the stated vaccine purpose, efficacy, impact, and source. The vaccine expert might find these objections to be dismissable. After all, these concerns might be addressed by simply looking at the impact of past vaccines, scientific studies of efficacy of the current one being promoted, and understanding of how the vaccine was created and how it works. This expert may try to address objections through the lens of their expertise, dismissing objections as being misinformed, illogical, or unreasoned. But, could work have been done at earlier stages to engage these future skeptical audiences? How might things have been different if these individuals and their respective lenses were part of early discussions? Would this have led to different approaches or technical solutions? We could consider the future skeptics to be the experts of their own experience, and this expertise to be a worthy lens to incorporate earlier in the process to ensure trust later on.

**FIGURE 2:**
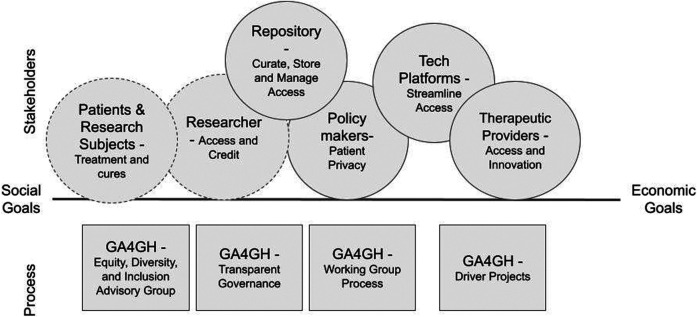
GA4GH has been a multi stakeholder initiative from its founding. It has built a reputation for cross-stakeholder engagement through transparent inclusive processes and is able to attract a broad range of stakeholders to participate in standards development and implementation.

GA4GH recognizes the importance of including diverse perspectives and lenses early in the standards-making process (Figure 2). In early 2020, the organization created an Equity, Diversity, and Inclusion Advisory Group for the express purpose to “find equitable and inclusive ways to bring diverse ideas into our standards creation process.” (GA4GH OmicsXchange, 2020) This group engages “intentional community” principles (Vogl, 2016) to consider who should be included in the standard process and intentionally build a community that includes these parties with the goal of ensuring input and buy-in.

#### 3.2.5 Including Diverse Stakeholders: The Expertise of End-Users

How can the trust built during the process of creating technical standards be transferred to the communities that will use the product but may not be privy to or understand the things unsaid—the norms, background, and implicit knowledge and understanding that something like a standard may contain?

Often with efforts that rely on a high degree of technical and subject matter expertise, the process of discovery and development goes something like this:

1. Identify and describe the problem
2. Develop hypotheses based on prior art, try out solutions, collaborate with other experts
3. Decide on a solution, often getting critiques and feedback from other experts
4. Disseminate more widely, sometimes creating descriptions or versions of the solution that are more accessible to other (non-expert) audiences

With this model, most end-users see only the packaged result. This is similar to back when regulated health and nutritional claims were not included on packaged products. (Institute of Medicine, 2010). Marketers might have provided terms like “healthy” or “good for you” as terms that someone who doesn’t produce packaged food should understand. But do these words help generate trust for the end-user who, say, is diabetic and needs to limit sugar intake, or has a food allergy? The end-user is an expert on how they will use the product and how their body might react to it, but words like “healthy” do not provide enough information for them to have trust that this product will not make them sick. The ingredients alone may not be enough. How it was prepared may be important for religious reasons (for example, keeping kosher), or for cultural reasons (for example, how its creation may have impacted the earth).

Food producers are getting better at engaging with end users to understand and include key factors in design, production, and marketing processes. It is no longer uncommon for packaged food labels to provide details about origin, method of creation, and ingredients, as well as values of those who are creating the product. The creation of things like technical standards, infrastructure, and research-based outcomes have not yet caught up. The omission of details for diverse audiences results from a lack of consideration for diverse stakeholder perspectives and needs. And these omissions impact trust.

GA4GH brings the end user into the standard process through Driver Projects. GA4GH Driver Projects are real-world genomic data initiatives that help guide development efforts and pilot the tools developed as part of the standards-making process. In addition, these projects enable stakeholders around the globe to advocate, mandate, implement and use GA4GH frameworks and standards in their local contexts, thereby building applicability and trust.

#### 3.2.6 Learnings

The Wednesday meeting of the GA4GH Data Use and Researcher Identity (DURI) work stream meeting is coming to a close. Trust has been built into their process in two key ways:

1. Inclusion of diverse voices early in the process: Through the inclusive community programs advocated for by GA4GH’s Equity, Diversity and Inclusion Advisory Group, early discussions include diverse voices. This practice enables a broad set of factors to be included for consideration, including religion, culture, relationship to the earth, socioeconomic status, logistics, and many others.
2. Encouraging practical use of standards as feedback input: Mechanisms such as the Driver Projects have been put in place to ensure that the solutions and their related benefits can be described using a broad set of lenses.

Including many broad perspectives from the beginning may slow the standards development process, but it will help pave the way for greater trust, use, and adoption of the standards that are developed.

### 3.3 Case Study: ORCID

Like Seamless Access and GA4GH’s DURI Project, ORCID started with a beastly technical problem, in this case, uniquely identifying each researcher in the world. ORCID took a researcher-centric approach to solving the problem, enshrining individual-level control and privacy into its foundational principles. It also built a multi stakeholder governance group and established bylaws before building any technology, establishing a reputation that was the foundation for creating legitimacy (Zeyen et al., 2016).

“To build community requires vigilant awareness of the work we must continually do to undermine all the socialization that leads us to behave in ways that perpetuate domination.”― bell hooks, Teaching Community: A Pedagogy of Hope.

**FIGURE 3:**
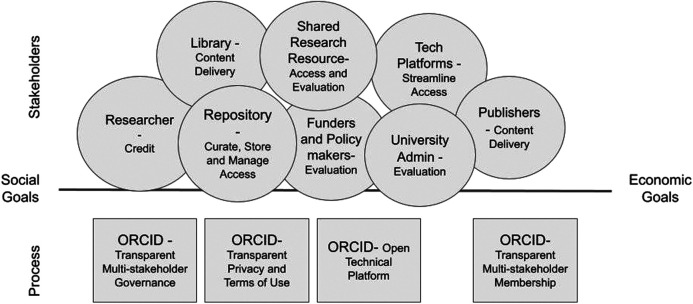
Like GA4GH, ORCID has been a multi stakeholder initiative from its founding. It started with a practical solution, and built adoption by engaging iteratively with stakeholders, developing trust through transparent processes and demonstrating value and mutual social and economic benefit.

With vision, principles, and governance established, ORCID could then transition to developing technology requirements in collaboration with its communities. And show by example, over and over again, that the organization adheres to its principles. This was a slower start than either SeamlessAccess or DURI, but it set the stage for cross-stakeholder communication at the outset, rather than trying to bring groups in later on. As ORCID developed its core technology, design decisions were driven by the fundamental ORCID tenets of community governance and researcher control. Articulated in ORCID’s Trust Program, it was the iterative experiences between and among stakeholder groups, the ORCID team, and the technology through which legitimacy and then trust emerged. Starting with early adopters, then focusing on publishers, research institutions, funders, and researchers, ORCID has demonstrated that it listens to and respects its stakeholders, and hews to its principles while evolving its offering as it learns more about community needs through working groups, community meetings, and consortia partners (Figure 3). Over the 10 years since the ORCID Board was founded, ORCID launched its registry, generated a base of over 10 million users, and on-boarded over 1,000 members in countries around the world, no small feat for a non-profit start-up.

In this section, we explore how ORCID has applied its trust framework in its work with its implementing partners, and how it has evolved with growing adoption.

#### 3.3.1 Launch Partners

As ORCID prepared to launch in 2012, we gathered a group of highly motivated partners to test our APIs and develop integrations that would be available when we launched the Registry (ORCID, 2020; Launch Part, 2020). We looked for recognizable platforms with broad researcher usage that were jazzed by ORCID’s mission and could invest resources and turn around a project in a short period of time. We listened, we learned, we launched.

We took an intentionally iterative approach to technology. As a start-up, we knew we couldn’t know everything at the beginning, and that we were likely to make mistakes. We needed the flexibility to re-group. We had regular meetings with our launch partners, and we also set up user forums to gather feedback, prioritize features, and address issues.

One of the early decisions we made was to err on the side of rapid integration and reduced user burden. An example of this in practice was the design decision to not require email verification during the ORCID registration process. Yes, I can see you wincing. It seemed like a good idea at the time because it brought more implementers to our door and streamlined the process of using ORCID for researchers in standard workflows. However, we found that when email verification was left out of the registration process, a substantial proportion of account emails were never verified. This had the unintentional consequence of increasing researcher burden because we had to send out multiple messages to request email verification, it led to numerous help desk tickets filed by researchers requesting access to their accounts, and it hindered our ability to track active records, a key indicator of researcher adoption.

Over the next 4 years, researchers started to preferentially use systems that had integrated ORCID. Stakeholder sentiment helped to encourage implementers to add email verification into their workflows, assuaging fears that the extra steps would cause researchers to use other platforms. In turn, in 2017, ORCID was able to require email verification in all ORCID integrations, as well as require it for researchers to access basic ORCID account features (Demain, 2017). This example of stakeholder alignment and researcher engagement shows how increased trust in a research infrastructure can enable iterative improvements and broaden adoption.

#### 3.3.2 API Versioning

Anyone involved in a technology start-up knows that driving early adoption is key. Without users, who really cares how cool the technology is. In the world of non-profits, the primary way to drive adoption is through mission alignment and mutual benefit. In our eagerness to onboard partners, we customized APIs to specific use cases. After 18 months, we were supporting over 20 API versions, which any developer will know is not sustainable. However, this approach at launch is not horrible, and is in fact quite common. Customization allowed us to work closely with our partners, figure out what worked well for users and what we could abstract across multiple platforms, and ultimately what features to fight for when developing the next API version. However, at some point, the customization needs to end and harmony must be established.

ORCID released its final mock API in March 2012 (ORCID, 2012). In addition to an ongoing API Users Group open to all with interest in the API, we formed a cross-stakeholder technical working group in February 2013 to examine the metadata used for Works in the ORCID Registry. The group helped us review the API and service models, and supported the iteration and socialization of a new API, version 1.2 (Paglione, 2013).

Over the next few years, we iterated on this API backbone model but knew we needed to make a break from initial assumptions to enable scalability. The ORCID record was not a monolithic document. We needed to enable calls and updates to individual sections and items. We launched version 2.0 in 2017, which helped manage hyper-authored publications, reduced confusion for implementers, and also added new functionality to support peer review recognition, improved user notifications, and the ability to support almost any persistent identifier (Peters, 2017).

We sunsetted version 1.2 at about the same time we started developing API v 3.0 (Teresa, 2018), and in 2020 transitioned to API 3.0 (Demain, 2020). Through this evolution, we worked closely with implementers to test new API functionality in early release candidates and also developed a policy for how API versioning would be handled so that enough time was given implementers to update and to ensure that priorities of implementers, members, and researchers were all considered in the versioning process (Blackburn, 2021).

API versioning is not unique to ORCID. However, how ORCID has handled versioning is an example of how to evolve technology while building trust across stakeholder groups. That ORCID was able to sustain, develop, and launch 3 substantially different APIs in its first 9 years is testament to the strength of the ORCID team and its commitment to serving ORCID communities.

#### 3.3.3 Implementation Documentation

As the ORCID user base grew, more stakeholders and more platforms began to implement ORCID features. Here again, ORCID took a broad approach, encouraging multiple approaches. We captured use cases specific to countries, community sectors, and workflows. The challenge came in sense-making for our implementers and users—and for our Help Desk. Just like the API, it is not sustainable to support custom documentation for every use case. We needed to consolidate messaging and documentation. It took several iterations to figure out which workflows worked best, decide what to prune, and then how to group use cases and information in a meaningful way.

Our Help Desk was launched in 2012, with live support, online documentation, and a User Forum with voting for new features. In 2014, we launched our first major documentation update, focused on implementers (Bryant, 2014). With more feedback from our growing member base, we created our Member Support Center in 2015, organizing technical documentation into sector-specific workflow guides, augmented by planning and communication resources (Paglione, 2015). We outgrew our user help desk system, and in 2018 with help from community translators transitioned to a new platform that enabled better local language support; along with this we also released more standardized help documentation and videos and were able to capture better statistics on how well we were serving our users (Cardoso, 2018).

The latest and for sure not the last documentation iteration was a massive upgrade to the ORCID Website in 2020, which updated how content was organized, based on usage patterns and community consultation. (Petro, 2019). In turn, these changes have both enabled and supported a pro-active product approach that more seamlessly integrates user feedback and key statistics to track ORCID adoption and impact. (Demain et al., 2021). Again, the iterative approach has allowed ORCID to engage stakeholders, test new approaches, integrate what works, and continue to innovate and build trusted services to meet the evolving needs of the ORCID community.

#### 3.3.4 Certification of ORCID Service Providers

Throughout these product iterations, ORCID sought to ensure stakeholder alignment by keeping track of its members and implementers: who is using what API version for which use case(s). Similarly, ORCID continues working to engage researchers by streamlining the user experience and clarifying the benefits of using ORCID in research workflows.

With more platforms integrated, this community management work becomes more difficult, but also easier. Compared to when it launched in 2012, ORCID in 2021 is much more visible in the community. With this visibility comes opportunity.

We learned from our initial attempt at creating an implementer community, Collect and Connect, what worked and what did not (Mejias, 2018). We brought together our core principles of community governance and researcher control to develop an enticing change management program for implementers. We engaged our community of implementers in developing a certification program (ORCID Senior Team, 2020), something we had talked about at launch but decided was not the right time.

Now, within 1 year of its launch, the certification program has certified 15 platforms, with global reach (ORCID). One of the goals of this program is to drive a common researcher-centric user experience, another is to recognize certified implementers. The program meets ORCID principles, provides a mutual benefit to our stakeholders, and strengthens ORCID engagement with implementers, providing a dedicated channel for updates on new developments and listening to implementer experiences and feedback. And, the certification program increases trust among those using ORCID by extending the principles and values of ORCID to integrations that build on the ORCID platform.

#### 3.3.5 Learnings

Since 2012, ORCID has evolved from a nimble and high-energy organization to one that is established, sustainable, and influential. It has done this through enthusiastic stakeholder engagement and patient community development. By respecting researchers. By partnering with its stakeholders to try many approaches and learning from mistakes. By always centering on its core values and principles and keeping its activities mission focused, even when it makes some stakeholders feel their needs are not being met. This is what makes up ORCID’s trust network.

## 4 Conclusion

**FIGURE 4:**
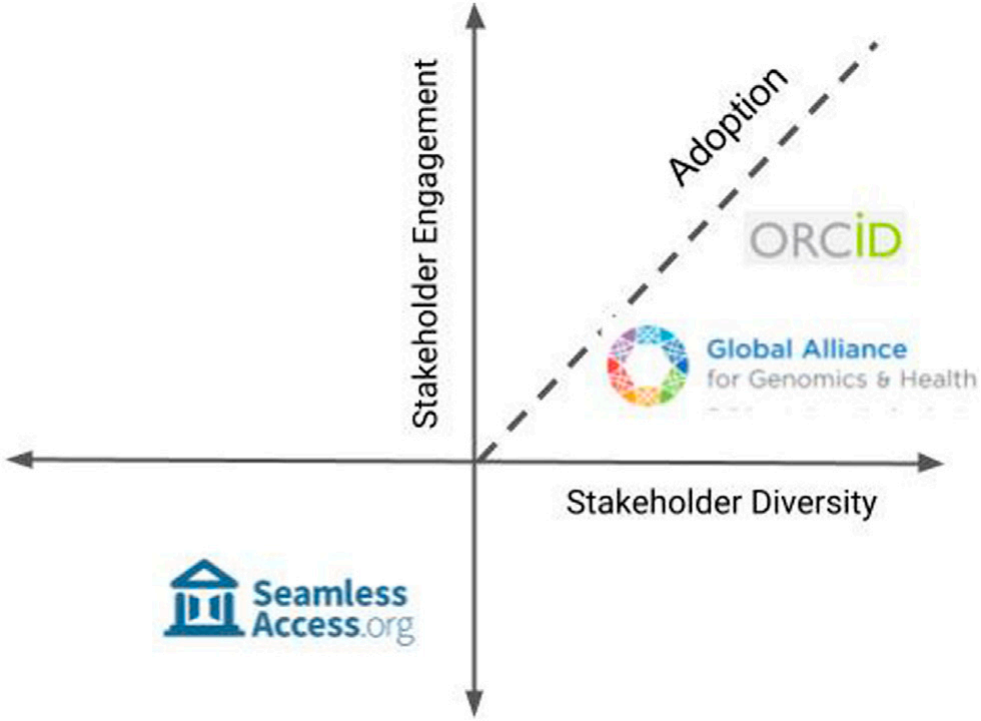
Adoption is driven by engagement of all stakeholders. Successful multi stakeholder initiatives motivate community participation through collective social and economic incentives.

Infrastructure is a critical component of research, whether it is manifested as technical standards, services, or community norms. Research infrastructure requires community trust for its adoption. As these case studies illustrate, we must take care to draw a wide circle when including stakeholders and interests in the design of infrastructure. We need to consider social and economic motivations and work to develop infrastructures are mutually beneficial. We also must ensure that there are transparent processes in place to support ongoing stakeholder engagement. Plotting the three infrastructures on axes of stakeholder diversity and engagement, we see these two factors can predict community adoption (Figure 4).

We must find ways to listen outside of our expertise and comfort zones, and build open, ethical, and socially responsible infrastructure through iterative community consultation. The concept of “connected professionalism”—where expert groups are more porous and consider societal perspectives—is relevant here (Noordegraaf, 2015). Infrastructure principles must be transparent; this is the foundation for open governance (Wiedenhoft et al., 2017). We must also consider how the infrastructure will be supported over time so that it may be adopted, adapted, and accessed. And we need to ensure that infrastructures are designed so that researchers—communities of experts, contributors, and users—can use and benefit from them. This finding is in line with work on sustainability of “platform as commons” through participatory design, such as we see for open source software (Poderi, 2019).

A compelling theoretical framework for multi-stakeholder initiatives combines club theory with institutional theory, and posits company interest in joining an initiative is largely based on reputational risk and reward (Zeyen et al., 2016). However, this framework is largely based on economic incentives for participation. Open infrastructure initiatives have an equal or stronger social good component; how then to drive participation and adoption? Here, stakeholder theory provides a means for both justifying and assessing engagement across economic, social, and other factors (Jamali, 2008). Normative stakeholder theory is rooted in the view that customers and firms share an environment, and holds that all stakeholders are intrinsically valuable and deserve consideration, whether or not they have a direct economic stake. In this view, individual researchers are not just targets for marketing initiatives to grow market share, they become as important as firms for the views and experiences they bring to design and implementation. Similarly, game theory shows that reciprocity (adoption) is driven by trust, which is in turn dependent upon the beliefs stakeholders have about other initiative participants (Cox, 2004). How engagement is constructed is critically important; bringing together individual “experts” rather than representatives can lead to stronger trust, suggesting that working groups may be a key component of infrastructure trust (Song, 2009). Multi stakeholder initiatives thus provide the venue to drive institutional change and create mutual benefit through inclusive participation by a range of stakeholders.

The case studies we present align with and support this theoretical framework, showing that successful multi stakeholder initiatives (success measured in terms of infrastructure or standards adoption) engage diverse individual actors as well as institutions. We argue that it is in balancing social and economic incentives that initiatives can attract both the institutions that can effect structural change, and the people who can drive this change through their participation and advocacy.

Trust is not that easy, and once it’s built, it’s not guaranteed to continue. Authority to adopt and control rests in a community. This means that ongoing and contextually meaningful outreach and engagement has to happen for infrastructures to maintain trust and provide community benefit. Items may seem out of scope to one stakeholder group, but we must be prepared to listen and address issues across a range of diverse perspectives. Ongoing working groups and a transparent governance structure are necessary for initiative evolution and sustainability.

We find intriguing parallels with co-production and community welfare initiatives, where the concept of “care” is paramount. The difference between “caring about” and “caring for” can have deep implications for stakeholder support and infrastructure sustainability (Light and Seravalli, 2019). Those initiatives that are designed to engender reciprocal accountability and mutual commitment also encourage reflexive engagement among stakeholders.

Infrastructures that succeed do so because the communities they serve care deeply about their success. Care deeply enough to take the time to take part in developing standards, building practice communities, and, in so doing, build the interpersonal and inter-stakeholder trust needed to implement global research infrastructures that can support broad participation, adoption, and benefits for public welfare.
